# Associations between dietary coenzyme Q10 intake and lipid profiles in adults: a national cross-sectional study

**DOI:** 10.3389/fnut.2024.1472002

**Published:** 2024-11-12

**Authors:** Mingzhu Zhao, Zezhong Tian, Dan Zhao, Huiying Kuang, Ying Liang, Zhihao Liu, Yixuan Xu, Shanshan Hou, Zepei Zhong, Yan Yang

**Affiliations:** ^1^School of Public Health (Shenzhen), Shenzhen Campus of Sun Yat-sen University, Shenzhen, China; ^2^Guangdong Engineering Technology Center of Nutrition Transformation, Sun Yat-sen University, Shenzhen, China; ^3^Guangdong Provincial Key Laboratory of Food, Nutrition and Health, Sun Yat-sen University, Guangzhou, China

**Keywords:** dietary coenzyme Q10, lipid profiles, dose–response relationship, cross-sectional study, Chinese adults

## Abstract

**Objective:**

This study aimed to determine the average intake of CoQ10 from dietary sources and explore the dose–response relationships between the dietary-derived CoQ10 intake and lipid profiles.

**Methods:**

We performed a cross-sectional study based on the China Health and Nutrition Survey, which included 7,938 adults. The dietary intake assessment used three consecutive 24-h recalls combined with a household inventory. Serum was used for lipid profiling.

**Results:**

The average dietary-derived CoQ10 intake was 5.4 mg/day in Chinese adults. The dietary CoQ10 intake of the highest quartile (Q4 ≥ 6.96 mg/day) was negatively associated with total cholesterol (TC) [−0.12 (−0.19, −0.06) mmol/L], low-density lipoprotein cholesterol (LDL-C) [−0.17 (−0.23, −0.10) mmol/L], and non-high-density lipoprotein cholesterol (non-HDL-C) [−0.12 (−0.18, −0.05) mmol/L], while positively associated with apolipoprotein A-1 (ApoA1) [0.10 (0.08, 0.13) g/L] and triglycerides (TG) [0.14 (0.05, 0.23) mmol/L], compared to the lowest quartile (Q1 < 1.88 mg/day). Besides, dietary CoQ10 intake showed nonlinear dose–response associations with the above lipid variables (all *P*_nonlinear_ < 0.05).

**Conclusion:**

Dietary-derived CoQ10 intake may be associated with some lipid profiles, such as TG, ApoA1, TC, LDL-C, and non-HDL-C. However, CoQ10 from dietary sources may not be a good choice for individuals who need to CoQ10 supplement.

## 1 Introduction

Cardiovascular diseases (CVDs) are the leading cause of death and disability worldwide (1, 2). Dyslipidemia is a modifiable and established risk factor for CVDs and is commonly found in people with obesity (3, 4). Dyslipidemia refers to the imbalance in the levels of one or more circulating lipids, including elevated total cholesterol (TC), low-density lipoprotein cholesterol (LDL-C), triglycerides (TG), and/or reduced high-density lipoprotein cholesterol (HDL-C). Dietary intervention is beneficial for human health and is also one of the effective strategies to improve dyslipidemia (5). Foods contain a variety of bioactive components that often provide multiple health benefits, such as antioxidative properties, lipid modulation, anti-inflammatory characteristics, antihypertensive attributes, etc., and may also help reduce the risk of CVDs (6–9).

Coenzyme Q10 (CoQ10) is an established lipid-soluble, antioxidative quinone that plays a vital role in the electron transport chain in the inner mitochondrial membrane and cell energy metabolism (10, 11). Commonly, endogenous synthesis of CoQ10 is sufficient, but exogenous CoQ10 supplementation from different foods and dietary supplements is necessary when CoQ10 is deficient in some diseases, such as dyslipidemia (12, 13). Many randomized controlled trials (RCTs) demonstrated that CoQ10 supplementation might benefit lipid profiles (14, 15).

Of note, previous trials focused on the effects of relatively high doses of CoQ10 supplementation, with a content of about 28–400 mg per capsule. CoQ10 is readily available in everyday foods, such as viscera, meat, fish, and nuts. However, the Chinese population’s mean intake of CoQ10 from dietary sources is unknown, and there was no study investigating the associations between dietary CoQ10 from daily foods and lipid profiles. Up to now, whether there are dose–response relationships between dietary CoQ10 intake and lipid profiles is also unknown. Furthermore, previous RCTs with ideal and controlled circumstances may make it hard to identify the potential regulatory factors that may affect the relationships between dietary CoQ10 and blood lipids, compared with observational studies in the real world.

Therefore, our study aimed to determine the average intake of CoQ10 in the daily diet and investigate the relationships between dietary CoQ10 intake and lipid profiles in general Chinese adults without a diagnosis of CVDs. Further, this study attempted to examine potential dose–response relationships between dietary CoQ10 and lipid profiles. We hypothesized that higher dietary CoQ10 intake would be associated with lipid profiles and that there would be dose–response relationships between dietary CoQ10 and lipid profiles.

## 2 Methods

### 2.1 Study design

This national cross-sectional study used a Chinese open cohort: the China Health and Nutrition Survey (CHNS) (16). The CHNS accumulated extensive information from 8 to 15 provinces (autonomous regions, municipalities) about community, family, individual, diet, physical examinations, and biochemical examinations and was, therefore, an appropriate tool for researching the Chinese population. All participants provided written consent before the survey. The CHNS was carried out following the Declaration of Helsinki, and its protocol was approved by the Ethics Committee of the University of North Carolina at Chapel Hill and the National Institute for Nutrition and Health, Chinese Center for Disease Control and Prevention (17). Further details of this survey were available in previous publications (18, 19).

### 2.2 Study population

**Figure 1:**
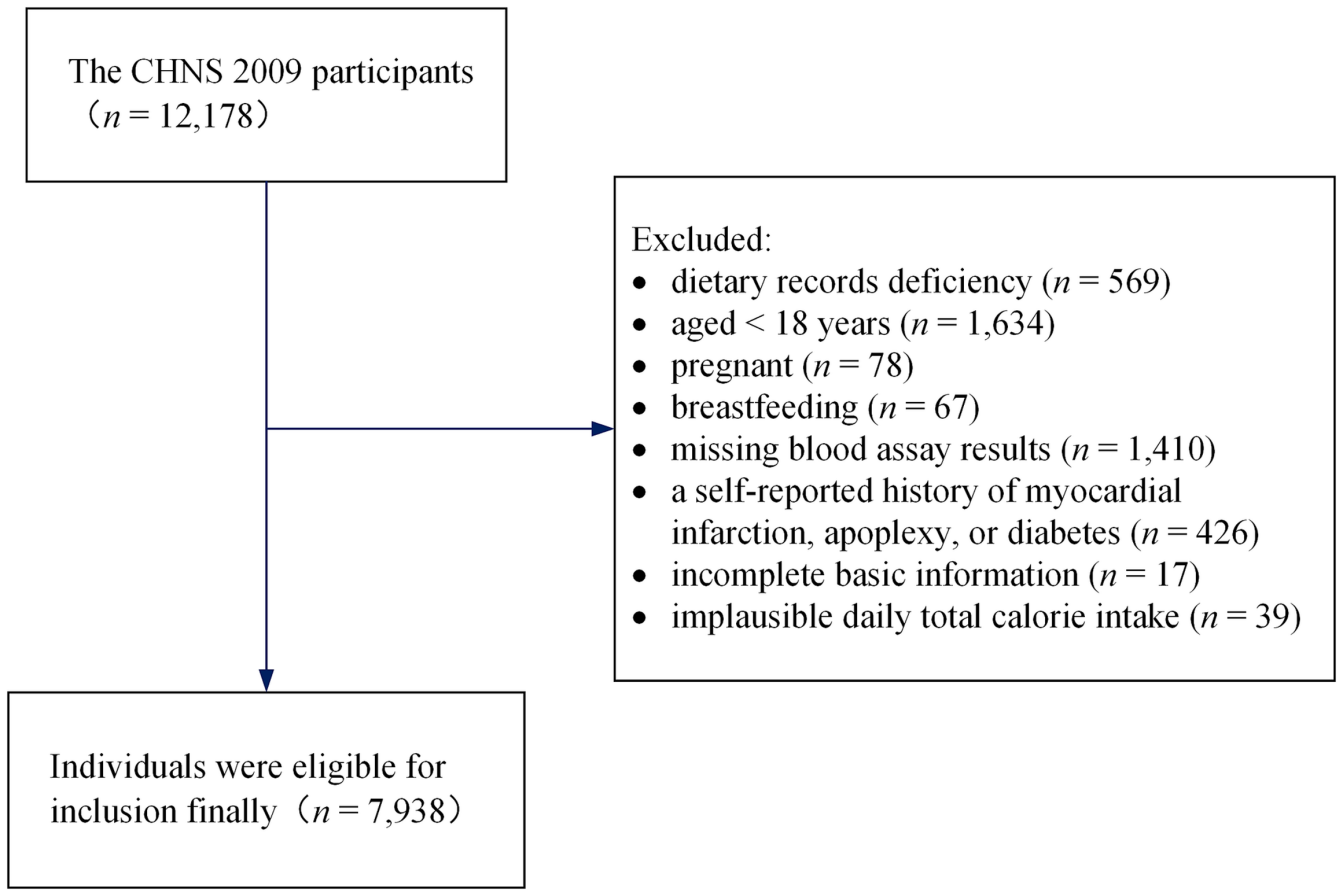
Selection criteria of the study samples.

The current study acquired data from the 2009 round of the CHNS. The publicly available dataset can be accessed directly on the Internet: https://www.cpc.unc.edu/projects/china. The CHNS 2009 covered 217 communities, including 12,178 individuals from 4,517 households in 9 provinces or autonomous regions with a considerable difference in geography, and it collected fasting blood in participants ≥7 years old to measure biomarkers for the first time. We excluded respondents who had dietary records deficiency (*n* = 569), aged <18 years at the time of the survey (*n* = 1,634), those who were pregnant (*n* = 78) or breastfeeding (*n* = 67), and those with missing blood assay results (*n* = 1,410). Additionally, respondents with a history of apoplexy, diabetes, or myocardial infarction (*n* = 426) were excluded since these conditions may alter dietary and lifestyle habits. We also excluded respondents with incomplete basic information (*n* = 17) and implausible daily total calorie intake (> 5,000 or < 700 kcal/day, *n* = 39) (20). Finally, 7,938 individuals were eligible for inclusion (3,737 men and 4,201 women) in the cross-sectional analysis, with ages ranging from 18 to 94 years. The screening process is shown in Figure 1.

### 2.3 Dietary intake assessment

The assessment was conducted using a household inventory obtained from households along with three consecutive 24-h dietary recalls obtained from individuals to estimate dietary intake more accurately (21). In each sampling unit, three consecutive days were equally and randomly selected to assess dietary intake. In addition to the inventory of all foods at the start and end of the 3 days, we also recorded the food purchased, grown, or discarded by each household and the number of person-days during this period (22). The usage of oil and condiments (such as salt, sugar, and sauces) was also estimated according to household dietary data (23). Individual records of daily food, mainly including ingredients, amount, meal location, and cooking method for all food consumption (involved in staple foods, side dishes, snacks, fruits, and beverages), were accomplished using food models and picture aids by trained and experienced interviewers (22). A quality check was performed by comparing the daily dietary consumption based on a household inventory with same-day dietary intake based on 24-h recalls (24, 25).

The calculation of total energy intake was based on previous studies (26, 27). Also, consumption of various foods was obtained with the aid of China Food Composition Tables and then further converted to daily dietary intake of CoQ10 using a previous study that provided an overview of CoQ10 contents in various foods (28–30). The participants were categorized into quartiles as Q1, Q2, Q3, and Q4 according to dietary CoQ10 intake levels in this study.

### 2.4 Lipid profiles assessment

The serum samples were collected as previously described (31). All samples were tested using Hitachi 7,600 automated analyzer (Hitachi Inc., Tokyo, Japan) in the Beijing central laboratory of China-Japan Friendship Hospital, Ministry of Health (medical laboratory accreditation certificate ISO 15189:2007) with strict quality control (31). TC, TG, HDL-C, and LDL-C were detected by an enzymatic method (Kyowa Medex Co., Ltd., Tokyo, Japan). Apolipoprotein B (ApoB) and apolipoprotein A-1 (ApoA1) were detected by diagnostic reagents (Randox Laboratories Ltd., United Kingdom), as well as lipoprotein(а) [Lp(a)] by diagnostic reagents (Denka Seiken Co., Ltd., Japan). Non-HDL-C was calculated as TC minus HDL-C. The diagnostic criteria for dyslipidemia were as follows (32): (1) TC ≥ 6.22 mmol/L (240 mg/dL) was considered as hypercholesterolemia; (2) TG ≥ 2.26 mmol/L (200 mg/dL) was considered as hypertriglyceridemia; (3) HDL-C < 1.04 mmol/L (40 mg/dL) was considered as low HDL-C; (4) LDL-C ≥ 4.14 mmol/L (160 mg/dL) was considered as high LDL-C.

### 2.5 Covariates assessment

A portable SECA stadiometer was used to obtain barefoot measurements of height with an accuracy of 0.1 cm, and a calibrated beam scale was used to measure barefoot weight in light clothing with an accuracy of 0.1 kg (31). Weight in kilograms divided by height in meters squared determines body mass index (BMI). The above biochemical and physical measurements were performed uniformly by qualified testers following pre-defined SOPs.

Demographics, health status, and tobacco and alcohol consumption were all collected via an adult questionnaire conducted by qualified investigators. Physical activity levels were assessed according to metabolic equivalent hours per week of occupation, household, leisure, and transportation (26, 33). Urbanization was measured using an urbanization scale with 12 components from the CHNS data (34). For further analysis, the provinces were categorized into the south and the north, bounded by the Qinling Mountain-Huaihe River Line.

### 2.6 Statistical analysis

Continuous variables were presented as mean ± standard deviation (SD), and categorical variables were presented as *n* (%). Differences between quartiles were examined with ANOVA for continuous variables and Chi-squared or exact Fisher test for categorical variables. General characteristics were described according to quartiles of dietary CoQ10 intake. Multiple linear regression models were used to investigate how dietary CoQ10 intake influenced lipid profiles (Lp(a), TC, TG, HDL-C, LDL-C, ApoA1, ApoB, and non-HDL-C) and adjusted for age, sex, daily total energy intake, smoking and drinking status, urbanization index, region, physical activity, education, and BMI. The results of linear regression were presented as beta (*β*) and 95% confidence intervals (CI) using the lowest quartile (Q1) as the reference group. Further, we explored the nonlinear relationships between dietary CoQ10 intake and lipid profiles via the restricted cubic spline (RCS) with 4 knots. Finally, subgroup analyses by sex (male, female), age (< 60 years, ≥ 60 years), and region (north, south) were carried out using the same covariates except the stratification variable.

In addition, we performed a sensitivity analysis to determine the robustness of the findings of multiple linear regression. Energy-adjusted dietary CoQ10 intake, calculated from the mean dietary CoQ10 intake plus the residuals from the regression of dietary CoQ10 intake on daily total energy intake, was used as a substitute for unadjusted dietary CoQ10 intake.

The data were analyzed using R version 4.1.3. *p* < 0.05 was considered significant.

## 3 Results

### 3.1 General characteristics

The general characteristics of 7,938 eligible participants by quartiles of CoQ10 intake from dietary sources were shown in Tables 1, 2 and Supplementary Table S1. The mean dietary CoQ10 intake for all participants was 5.4 mg/day, with a median [interquartile ranges] of 3.92 [1.88, 6.96] mg/day. Meat and meat products were the most important sources of dietary CoQ10, as shown in Supplementary Table S2. Moreover, the median [interquartile ranges] of dietary CoQ10 intake was 4.14 [2.10, 7.16] mg/day for men, 3.73 [1.73, 6.76] mg/day for women, 4.13 [2.06, 7.23] mg/day for people aged <60 years, 3.36 [1.48, 6.14] mg/day for people aged ≥60 years, 5.00 [1.93, 8.61] mg/day for northern people, and 3.54 [1.85, 5.87] mg/day for southern people. As shown in Supplementary Table S3, dietary CoQ10 intake varied significantly by sex, age, education, region, and BMI (all *p* < 0.05). The average age of participants was 49.9 years. 47.1% of the participants were male, and 26.1% were older persons (people aged ≥60 years). Furthermore, compared with the participants who had lower dietary CoQ10 intake, those with higher dietary CoQ10 intake were more likely to be younger, have a high proportion of males, have lower concentrations of TC, LDL-C, and non-HDL-C, have higher concentrations of TG and ApoA1 and have a higher intake of energy, carbohydrate, fat, and protein.

**Table 1:** Demographic information of participants in the CHNS by dietary CoQ10 intake.

Characteristics	All participants	Quartiles of dietary CoQ10 intake, mg/day				*p* value
		Q1 (< 1.88)	Q2 (1.88–3.92)	Q3 (3.92–6.96)	Q4 (≥ 6.96)	
Number of participants, *n*	7,938	1,984	1,989	1,981	1,984	
Dietary CoQ10, mg/day	5.4 ± 11.5	0.9 ± 0.6	2.9 ± 0.6	5.3 ± 0.9	12.6 ± 21.2	<0.001
Sex, *n* (%)						<0.001
Male	3,737 (47.1%)	837 (42.2%)	950 (47.8%)	960 (48.5%)	990 (49.9%)	
Female	4,201 (52.9%)	1,147 (57.8%)	1,039 (52.2%)	1,021 (51.5%)	994 (50.1%)	
Age, years	49.9 ± 14.8	52.2 ± 15.5	49.5 ± 14.8	49.3 ± 14.6	48.7 ± 14.1	<0.001
older persons, *n* (%)	2,074 (26.1%)	653 (32.9%)	518 (26.0%)	493 (24.9%)	410 (20.7%)	<0.001
BMI, kg/m^2^	23.3 ± 3.4	23.2 ± 3.5	23.0 ± 3.4	23.3 ± 3.3	23.6 ± 3.5	<0.001
Middle school or above, *n* (%)	4,506 (56.8%)	987 (49.7%)	1,168 (58.7%)	1,197 (60.4%)	1,154 (58.2%)	<0.001
Region, *n* (%)						<0.001
North	3,339 (42.1%)	816 (41.1%)	611 (30.7%)	734 (37.1%)	1,178 (59.4%)	
South	4,599 (57.9%)	1,168 (58.9%)	1,378 (69.3%)	1,247 (62.9%)	806 (40.6%)	
Residence, *n* (%)						<0.001
Urban	2,556 (32.2%)	611 (30.8%)	676 (34.0%)	690 (34.8%)	579 (29.2%)	
Rural	5,382 (67.8%)	1,373 (69.2%)	1,313 (66.0%)	1,291 (65.2%)	1,405 (70.8%)	
Urbanization index	67.0 ± 19.4	64.3 ± 19.5	69.2 ± 19.0	69.4 ± 19.2	65.1 ± 19.5	<0.001

Values were presented as mean ± standard deviation (SD) for continuous variables and n (%) for categorical variables. CoQ10, coenzyme Q10; BMI, body mass index.

**Table 2:** Lipid profiles and dyslipidemia status of participants in the CHNS by dietary CoQ10 intake.

Characteristics	All participants	Quartiles of dietary CoQ10 intake, mg/day				*p* value
		Q1 (< 1.88)	Q2 (1.88–3.92)	Q3 (3.92–6.96)	Q4 (≥ 6.96)	
Lp (a), mg/L	152.8 ± 219.2	157.3 ± 198.8	148.5 ± 204.0	159.2 ± 223.0	146.2 ± 247.6	0.167
TC, mmol/L	4.9 ± 1.0	4.9 ± 1.0	4.9 ± 1.0	4.8 ± 1.0	4.8 ± 1.0	<0.001
TG, mmol/L	1.6 ± 1.5	1.6 ± 1.2	1.7 ± 1.5	1.7 ± 1.5	1.7 ± 1.6	0.002
HDL-C, mmol/L	1.4 ± 0.5	1.5 ± 0.5	1.4 ± 0.4	1.5 ± 0.5	1.4 ± 0.5	0.154
LDL-C, mmol/L	3.0 ± 1.0	3.0 ± 1.0	3.0 ± 1.0	3.0 ± 1.0	2.9 ± 1.0	<0.001
ApoA1, g/L	1.2 ± 0.4	1.1 ± 0.3	1.1 ± 0.3	1.2 ± 0.4	1.2 ± 0.5	<0.001
ApoB, g/L	0.9 ± 0.3	0.9 ± 0.3	0.9 ± 0.3	0.9 ± 0.3	0.9 ± 0.3	0.898
Non-HDL-C, mmol/L	3.4 ± 1.0	3.5 ± 1.0	3.5 ± 1.0	3.4 ± 1.1	3.4 ± 1.0	0.002
Dyslipidemia, *n* (%)	2,651 (33.4%)	635 (32.0%)	687 (34.5%)	660 (33.3%)	669 (33.7%)	0.393
Hypercholesterolemia, *n* (%)	712 (9.0%)	188 (9.5%)	196 (9.9%)	173 (8.7%)	155 (7.8%)	0.116
Hypertriglyceridemia, *n* (%)	1,505 (19.0%)	346 (17.4%)	374 (18.8%)	379 (19.1%)	406 (20.5%)	0.113
High LDL-C, *n* (%)	821 (10.3%)	218 (11.0%)	227 (11.4%)	201 (10.2%)	175 (8.8%)	0.039
Low HDL-C, *n* (%)	1,013 (12.8%)	230 (11.6%)	268 (13.5%)	241 (12.2%)	274 (13.8%)	0.116

Values were presented as mean ± standard deviation (SD) for continuous variables and n (%) for categorical variables. CoQ10, coenzyme Q10; Lp(a), lipoprotein(а); TC, total cholesterol; TG, triglycerides; HDL-C, high-density lipoprotein cholesterol; LDL-C, low-density lipoprotein cholesterol; ApoA1, apolipoprotein A-1; ApoB, apolipoprotein B.

### 3.2 Linear associations between dietary CoQ10 intake and lipid profiles

The results of linear regression analyses in all eligible participants that examined how dietary CoQ10 intake influenced lipid profiles are shown in Table 3. In the adjusted model 2, the quartiles of dietary CoQ10 intake had significant associations with TC, TG, LDL-C, ApoA1, and non-HDL-C (all *P*_trend_ < 0.05). To be specific, compared with participants who had the lowest quartile (Q1 < 1.88 mg/day) of dietary CoQ10 intake, those who had the highest quartile (Q4 ≥ 6.96 mg/day) showed significantly higher levels of TG (*β* = 0.14 mmol/L, 95% CI: 0.05 to 0.23 mmol/L, *p* = 0.002) and ApoA1 (*β* = 0.10 g/L, 95% CI: 0.08 to 0.13 g/L, *p* < 0.001) and significantly lower levels of TC (*β* = −0.12 mmol/L, 95% CI: −0.19 to −0.06 mmol/L, *p* < 0.001), LDL-C (*β* = −0.17 mmol/L, 95% CI: −0.23 to −0.10 mmol/L, *p* < 0.001), and non-HDL-C (*β* = −0.12 mmol/L, 95% CI: −0.18 to −0.05 mmol/L, *p* < 0.001). There were consistent results when using energy-adjusted dietary CoQ10 intake (Supplementary Table S4).

**Table 3:** Linear regression associations between the quartiles of dietary CoQ10 intake and lipid profiles in CHNS.

	Crude model			Adjusted model 1			Adjusted model 2		
	*β* (95% CI)	*p* value	*p*_trend_	*β* (95% CI)	*p* value	*p*_trend_	*β* (95% CI)	*p* value	*p*_trend_
Lp(a), mg/L									
Q1 (< 1.88)	Ref		0.305	Ref		0.459	Ref		0.449
Q2 (1.88–3.92)	−8.73 (−22.36, 4.90)	0.209		−3.25 (−17.01, 10.51)	0.643		−4.15 (−18.06, 9.75)	0.558	
Q3 (3.92–6.96)	1.93 (−11.72, 15.57)	0.782		6.85 (−7.02, 20.72)	0.333		4.93 (−9.08, 18.94)	0.490	
Q4 (≥ 6.96)	−11.08 (−24.72, 2.56)	0.111		−9.44 (−23.75, 4.87)	0.196		−9.19 (−23.62, 5.24)	0.212	
TC, mmol/L									
Q1 (< 1.88)	Ref		< 0.001	Ref		< 0.001	Ref		< 0.001
Q2 (1.88–3.92)	−0.01 (−0.08, 0.05)	0.643		0 (−0.06, 0.06)	0.955		0 (−0.06, 0.06)	0.891	
Q3 (3.92–6.96)	−0.06 (−0.13, 0)	0.047		−0.06 (−0.12, 0.01)	0.076		−0.06 (−0.12, 0)	0.042	
Q4 (≥ 6.96)	−0.13 (−0.19, −0.07)	< 0.001		−0.12 (−0.18, −0.06)	< 0.001		−0.12 (−0.19, −0.06)	< 0.001	
TG, mmol/L									
Q1 (< 1.88)	Ref		< 0.001	Ref		0.003	Ref		0.006
Q2 (1.88–3.92)	0.09 (0, 0.18)	0.047		0.07 (−0.02, 0.16)	0.140		0.10 (0.01, 0.18)	0.034	
Q3 (3.92–6.96)	0.09 (0, 0.18)	0.041		0.06 (−0.03, 0.16)	0.169		0.07 (−0.01, 0.16)	0.101	
Q4 (≥ 6.96)	0.18 (0.09, 0.27)	< 0.001		0.15 (0.06, 0.25)	0.002		0.14 (0.05, 0.23)	0.002	
HDL-C, mmol/L									
Q1 (< 1.88)	Ref		0.159	Ref		0.761	Ref		0.957
Q2 (1.88–3.92)	−0.02 (−0.05, 0.01)	0.180		−0.01 (−0.04, 0.02)	0.614		−0.01 (−0.04, 0.02)	0.397	
Q3 (3.92–6.96)	0 (−0.03, 0.03)	0.921		0.01 (−0.02, 0.04)	0.363		0.01 (−0.02, 0.04)	0.415	
Q4 (≥ 6.96)	−0.03 (−0.06, 0)	0.058		−0.01 (−0.04, 0.02)	0.415		−0.01 (−0.04, 0.02)	0.616	
LDL-C, mmol/L									
Q1 (< 1.88)	Ref		< 0.001	Ref		< 0.001	Ref		< 0.001
Q2 (1.88–3.92)	−0.06 (−0.12, 0)	0.050		−0.04 (−0.10, 0.02)	0.228		−0.04 (−0.10, 0.02)	0.162	
Q3 (3.92–6.96)	−0.09 (−0.16, −0.03)	0.002		−0.09 (−0.15, −0.03)	0.005		−0.10 (−0.16, −0.04)	0.002	
Q4 (≥ 6.96)	−0.15 (−0.21, −0.09)	< 0.001		−0.16 (−0.23, −0.10)	< 0.001		−0.17 (−0.23, −0.10)	< 0.001	
ApoA1, g/L									
Q1 (< 1.88)	Ref		< 0.001	Ref		< 0.001	Ref		< 0.001
Q2 (1.88–3.92)	0.02 (0, 0.04)	0.120		0.02 (0, 0.04)	0.078		0.02 (−0.01, 0.04)	0.160	
Q3 (3.92–6.96)	0.07 (0.04, 0.09)	< 0.001		0.07 (0.05, 0.09)	< 0.001		0.07 (0.05, 0.09)	< 0.001	
Q4 (≥ 6.96)	0.09 (0.06, 0.11)	< 0.001		0.10 (0.08, 0.13)	< 0.001		0.10 (0.08, 0.13)	< 0.001	
ApoB, g/L									
Q1 (< 1.88)	Ref		0.782	Ref		0.644	Ref		0.512
Q2 (1.88–3.92)	0 (−0.01, 0.02)	0.648		0.01 (−0.01, 0.02)	0.366		0.01 (−0.01, 0.02)	0.249	
Q3 (3.92–6.96)	0.01 (−0.01, 0.02)	0.469		0 (−0.01, 0.02)	0.575		0 (−0.01, 0.02)	0.685	
Q4 (≥ 6.96)	0 (−0.01, 0.02)	0.839		0 (−0.02, 0.01)	0.680		0 (−0.02, 0.01)	0.632	
Non-HDL-C, mmol/L									
Q1 (< 1.88)	Ref		< 0.001	Ref		< 0.001	Ref		< 0.001
Q2 (1.88–3.92)	0.01 (−0.06, 0.07)	0.868		0.01 (−0.05, 0.07)	0.768		0.02 (−0.04, 0.08)	0.596	
Q3 (3.92–6.96)	−0.06 (−0.13, 0)	0.053		−0.07 (−0.13, −0.01)	0.028		−0.08 (−0.14, −0.02)	0.014	
Q4 (≥ 6.96)	−0.10 (−0.17, −0.04)	0.002		−0.11 (−0.17, −0.04)	0.001		−0.12 (−0.18, −0.05)	< 0.001	

Values were presented as beta (β) and 95% confidence intervals (CI). Adjusted model 1 adjusted for age, sex, daily total energy intake, smoking and drinking status, urbanization index, region, physical activity, and education. Adjusted model 2 further adjusted for BMI. CoQ10, coenzyme Q10; Lp(a), lipoprotein(а); TC, total cholesterol; TG, triglycerides; HDL-C, high-density lipoprotein cholesterol; LDL-C, low-density lipoprotein cholesterol; ApoA1, apolipoprotein A-1; ApoB, apolipoprotein B; BMI, body mass index.

### 3.3 Dose–response relationships between dietary CoQ10 intake and lipid profiles

**Figure 2:**
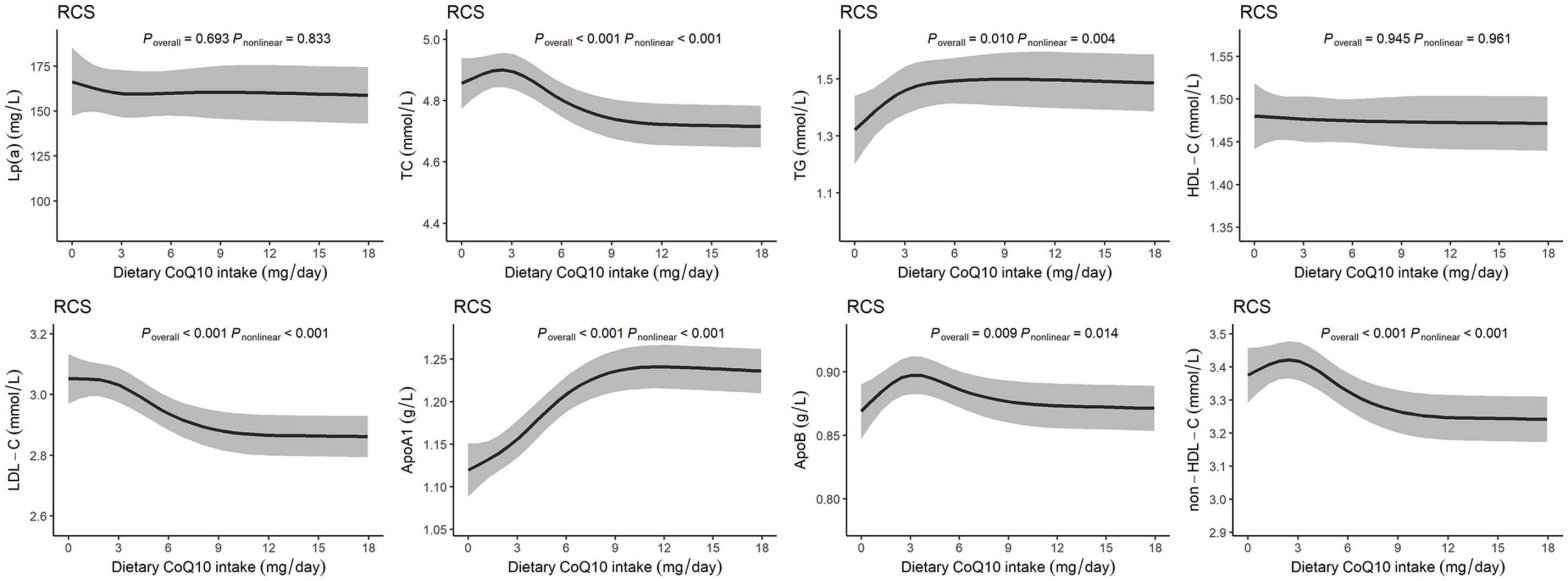
Dose–response relationships between dietary CoQ10 intake and lipid profiles. The RCS models with 4 knots showed the potential nonlinear dose–response relationships between dietary CoQ10 intake and Lp(a), TC, TG, HDL-C, LDL-C, ApoA1, ApoB, and non-HDL-C, adjusted for age, sex, daily total energy intake, smoking and drinking status, urbanization index, region, physical activity, education, and BMI.

Figure 2 shows the nonlinear dose–response relationships between dietary CoQ10 intake and lipid profiles. Most of the lipid profiles displayed nonlinear relationships with dietary CoQ10 intake (*P*_nonlinear_ < 0.05), except Lp(a) and HDL-C. There was a slight rise of TC, ApoB, and non-HDL-C within the lower level of dietary CoQ10 intake, which peaked at around 3 mg/day of CoQ10 and then reduced with increasing dietary CoQ10 intake. With increased dietary CoQ10 intake, TG and ApoA1 increased continuously and gradually stabilized. A sustained reduction was observed for LDL-C with increased dietary CoQ10 intake and then stabilized.

### 3.4 Subgroup analyses by potential effect modifiers

Subgroup analyses compared participants stratified by sex, age, and region (Tables 4–6). The associations between dietary CoQ10 intake and Lp(a), TC, HDL-C, LDL-C, ApoA1, ApoB, and non-HDL-C showed similar trends in different subgroups. In addition, the positive associations between dietary CoQ10 intake and TG were considered statistically significant only among women (Q4: *β* = 0.16 mmol/L, 95% CI: 0.05 to 0.26 mmol/L, *P*_trend_ = 0.006), participants aged <60 years (Q4: *β* = 0.18 mmol/L, 95% CI: 0.07 to 0.29 mmol/L, *P*_trend_ = 0.003), and participants living in the north of China (Q4: *β* = 0.26 mmol/L, 95% CI: 0.12 to 0.41 mmol/L, *P*_trend_ < 0.001).

**Table 4:** Subgroup analyses of linear regression associations between the quartiles of dietary CoQ10 intake and lipid profiles by sex in CHNS.

	Quartiles of dietary CoQ10 intake, mg/day				*p*_trend_
	Q1 (< 1.88)	Q2 (1.88–3.92)	Q3 (3.92–6.96)	Q4 (≥ 6.96)	
Lp(a), mg/L					
Sex, male	Ref	−7.23 (−25.26, 10.79)	0.31 (−17.80, 18.41)	−7.18 (−25.65, 11.29)	0.654
Sex, female	Ref	−2.01 (−22.72, 18.71)	8.42 (−12.54, 29.39)	−11.08 (−32.91, 10.75)	0.565
TC, mmol/L					
Sex, male	Ref	0.04 (−0.05, 0.13)	−0.04 (−0.13, 0.05)	−0.13 (−0.22, −0.04)	0.001
Sex, female	Ref	−0.02 (−0.10, 0.06)	−0.08 (−0.16, 0)	−0.10 (−0.19, −0.01)	0.010
TG, mmol/L					
Sex, male	Ref	0.13 (−0.03, 0.28)	0.09 (−0.06, 0.24)	0.14 (−0.02, 0.29)	0.140
Sex, female	Ref	0.07 (−0.03, 0.17)	0.06 (−0.04, 0.17)	0.16 (0.05, 0.26)	0.006
HDL-C, mmol/L					
Sex, male	Ref	0 (−0.05, 0.04)	0.03 (−0.01, 0.07)	0.03 (−0.02, 0.07)	0.103
Sex, female	Ref	−0.02 (−0.05, 0.02)	0 (−0.04, 0.04)	−0.04 (−0.07, 0)	0.154
LDL-C, mmol/L					
Sex, male	Ref	−0.02 (−0.12, 0.07)	−0.08 (−0.17, 0.01)	−0.19 (−0.28, −0.10)	< 0.001
Sex, female	Ref	−0.06 (−0.14, 0.02)	−0.11 (−0.19, −0.03)	−0.13 (−0.21, −0.05)	< 0.001
ApoA1, g/L					
Sex, male	Ref	0.03 (−0.01, 0.07)	0.10 (0.07, 0.14)	0.11 (0.07, 0.15)	< 0.001
Sex, female	Ref	0.01 (−0.02, 0.04)	0.04 (0.01, 0.07)	0.10 (0.07, 0.13)	< 0.001
ApoB, g/L					
Sex, male	Ref	0.01 (−0.01, 0.04)	0.01 (−0.02, 0.03)	0 (−0.03, 0.02)	0.542
Sex, female	Ref	0.01 (−0.02, 0.03)	0 (−0.02, 0.02)	0 (−0.02, 0.02)	0.937
Non-HDL-C, mmol/L					
Sex, male	Ref	0.04 (−0.05, 0.13)	−0.07 (−0.16, 0.02)	−0.16 (−0.25, −0.07)	< 0.001
Sex, female	Ref	0 (−0.09, 0.08)	−0.08 (−0.16, 0)	−0.06 (−0.15, 0.02)	0.049

Values were presented as beta (β) and 95% confidence intervals (CI). Adjusted for age, daily total energy intake, smoking and drinking status, urbanization index, region, physical activity, education, and BMI. CoQ10, coenzyme Q10; Lp(a), lipoprotein(а); TC, total cholesterol; TG, triglycerides; HDL-C, high-density lipoprotein cholesterol; LDL-C, low-density lipoprotein cholesterol; ApoA1, apolipoprotein A-1; ApoB, apolipoprotein B; BMI, body mass index.

**Table 5:** Subgroup analyses of linear regression associations between the quartiles of dietary CoQ10 intake and lipid profiles by age in CHNS.

	Quartiles of dietary CoQ10 intake, mg/day				*P*_trend_
	Q1 (< 1.88)	Q2 (1.88–3.92)	Q3 (3.92–6.96)	Q4 (≥ 6.96)	
Lp(a), mg/L					
Age, < 60 years	Ref	−2.83 (−19.57, 13.91)	6.80 (−10.00, 23.61)	−7.46 (−24.42, 9.50)	0.631
Age, ≥ 60 years	Ref	−6.42 (−31.47, 18.63)	1.03 (−24.53, 26.60)	−11.54 (−39.68, 16.59)	0.582
TC, mmol/L					
Age, < 60 years	Ref	−0.03 (−0.11, 0.04)	−0.08 (−0.16, −0.01)	−0.13 (−0.20, −0.05)	<0.001
Age, ≥ 60 years	Ref	0.07 (−0.05, 0.18)	−0.04 (−0.16, 0.08)	−0.14 (−0.27, −0.01)	0.024
TG, mmol/L					
Age, < 60 years	Ref	0.11 (0, 0.22)	0.11 (0, 0.22)	0.18 (0.07, 0.29)	0.003
Age, ≥ 60 years	Ref	0.07 (−0.06, 0.21)	0 (−0.14, 0.13)	0.01 (−0.14, 0.16)	0.954
HDL-C, mmol/L					
Age, < 60 years	Ref	−0.01 (−0.05, 0.02)	0 (−0.03, 0.04)	−0.01 (−0.05, 0.02)	0.666
Age, ≥ 60 years	Ref	−0.02 (−0.08, 0.03)	0.03 (−0.03, 0.08)	0.01 (−0.06, 0.07)	0.506
LDL-C, mmol/L					
Age, < 60 years	Ref	−0.07 (−0.14, 0)	−0.13 (−0.20, −0.06)	−0.18 (−0.25, −0.11)	<0.001
Age, ≥ 60 years	Ref	−0.01 (−0.13, 0.11)	−0.04 (−0.16, 0.08)	−0.15 (−0.28, −0.01)	0.040
ApoA1, g/L					
Age, < 60 years	Ref	0.01 (−0.01, 0.04)	0.06 (0.03, 0.08)	0.09 (0.07, 0.12)	<0.001
Age, ≥ 60 years	Ref	0.01 (−0.05, 0.07)	0.11 (0.05, 0.17)	0.13 (0.06, 0.19)	<0.001
ApoB, g/L					
Age, < 60 years	Ref	0.01 (−0.01, 0.03)	0 (−0.02, 0.02)	0 (−0.02, 0.02)	0.895
Age, ≥ 60 years	Ref	0.01 (−0.02, 0.04)	0 (−0.03, 0.03)	−0.02 (−0.05, 0.01)	0.276
Non-HDL-C, mmol/L					
Age, < 60 years	Ref	−0.02 (−0.09, 0.05)	−0.09 (−0.16, −0.02)	−0.12 (−0.19, −0.04)	<0.001
Age, ≥ 60 years	Ref	0.09 (−0.03, 0.21)	−0.07 (−0.19, 0.05)	−0.14 (−0.27, −0.01)	0.012

Values were presented as beta (β) and 95% confidence intervals (CI). Adjusted for sex, daily total energy intake, smoking and drinking status, urbanization index, region, physical activity, education, and BMI. CoQ10, coenzyme Q10; Lp(a), lipoprotein(а); TC, total cholesterol; TG, triglycerides; HDL-C, high-density lipoprotein cholesterol; LDL-C, low-density lipoprotein cholesterol; ApoA1, apolipoprotein A-1; ApoB, apolipoprotein B; BMI, body mass index.

**Table 6:** Subgroup analyses of linear regression associations between the quartiles of dietary CoQ10 intake and lipid profiles by region in CHNS.

	Quartiles of dietary CoQ10 intake, mg/day				*P*_trend_
	Q1 (< 1.88)	Q2 (1.88–3.92)	Q3 (3.92–6.96)	Q4 (≥ 6.96)	
Lp(a), mg/L					
Region, north	Ref	−7.76 (−31.59, 16.06)	5.07 (−17.53, 27.66)	−15.32 (−36.08, 5.43)	0.238
Region, south	Ref	−2.96 (−20.18, 14.25)	5.85 (−12.21, 23.90)	−3.89 (−24.61, 16.83)	0.955
TC, mmol/L					
Region, north	Ref	−0.01 (−0.11, 0.09)	−0.07 (−0.17, 0.02)	−0.11 (−0.20, −0.02)	0.008
Region, south	Ref	0 (−0.07, 0.08)	−0.06 (−0.14, 0.02)	−0.14 (−0.23, −0.05)	<0.001
TG, mmol/L					
Region, north	Ref	0.17 (0, 0.33)	0.17 (0.02, 0.33)	0.26 (0.12, 0.41)	<0.001
Region, south	Ref	0.03 (−0.07, 0.13)	0 (−0.11, 0.10)	−0.01 (−0.13, 0.11)	0.761
HDL-C, mmol/L					
Region, north	Ref	0.02 (−0.03, 0.07)	0 (−0.05, 0.05)	−0.01 (−0.06, 0.03)	0.420
Region, south	Ref	−0.02 (−0.06, 0.01)	0.02 (−0.01, 0.05)	0.01 (−0.03, 0.05)	0.173
LDL-C, mmol/L					
Region, north	Ref	−0.13 (−0.23, −0.03)	−0.15 (−0.25, −0.05)	−0.20 (−0.29, −0.11)	<0.001
Region, south	Ref	0 (−0.07, 0.08)	−0.06 (−0.14, 0.01)	−0.14 (−0.23, −0.05)	<0.001
ApoA1, g/L					
Region, north	Ref	0.02 (−0.03, 0.06)	0.13 (0.09, 0.18)	0.15 (0.11, 0.19)	<0.001
Region, south	Ref	0 (−0.02, 0.03)	0.03 (0, 0.05)	0.06 (0.03, 0.09)	<0.001
ApoB, g/L					
Region, north	Ref	0.01 (−0.02, 0.04)	0.02 (0, 0.05)	0 (−0.02, 0.03)	0.593
Region, south	Ref	0 (−0.01, 0.02)	−0.01 (−0.03, 0.01)	−0.01 (−0.04, 0.01)	0.131
Non-HDL-C, mmol/L					
Region, north	Ref	−0.03 (−0.13, 0.08)	−0.08 (−0.18, 0.02)	−0.09 (−0.19, 0)	0.031
Region, south	Ref	0.03 (−0.04, 0.10)	−0.08 (−0.16, −0.01)	−0.15 (−0.24, −0.06)	<0.001

Values were presented as beta (β) and 95% confidence intervals (CI). Adjusted for age, sex, daily total energy intake, smoking and drinking status, urbanization index, physical activity, education, and BMI. CoQ10, coenzyme Q10; Lp(a), lipoprotein(а); TC, total cholesterol; TG, triglycerides; HDL-C, high-density lipoprotein cholesterol; LDL-C, low-density lipoprotein cholesterol; ApoA1, apolipoprotein A-1; ApoB, apolipoprotein B; BMI, body mass index.

## 4 Discussion

In the present cross-sectional study, we evaluated the average intake of CoQ10 from the daily diet for the first time and found that the mean dietary CoQ10 intake was 5.4 mg/day in Chinese adults. The results of the present study indicated that dietary CoQ10 intake was positively associated with ApoA1 while negatively associated with TC, LDL-C, and non-HDL-C in the general population. However, it was unexpected to note that the dietary CoQ10 intake showed a positive association with TG. The RCS curves showed that dietary CoQ10 consumption has nonlinear dose–response relationships with TC, TG, LDL-C, ApoA1, ApoB, and non-HDL-C. Besides, subgroup analyses revealed that the associations between dietary CoQ10 intake and TG were heterogeneous among subgroups stratified by sex, age, and region.

We estimated the mean intake of CoQ10 in the Chinese population for the first time using the CHNS data. The findings of current research showed that the mean dietary CoQ10 intake was 5.4 mg/day, which was close to previous results in Denmark (3–5 mg/day) (35), Finland (5.4 mg/day for men and 3.8 mg/day for women) (36) and Japan (4.48 mg/day for total CoQ10) (37).

Consistent with the results of the present study, Zhang et al. found that CoQ10 supplementation (120 mg/day) for 24 weeks regulated lipid profiles in dyslipidemic individuals by increasing ApoA1 and reducing LDL-C and non-HDL-C (14). In line with our findings, Derosa et al. showed that those treated with CoQ10 (100 mg/day) for 3 months had lower TC and LDL-C in dyslipidemic subjects intolerant to statins (38). Meanwhile, a meta-analysis showed that patients with coronary artery disease who took CoQ10 had lower TC and higher HDL-C values (39). To our knowledge, no cross-sectional studies investigated the relationships between dietary CoQ10 intake and lipid profiles. Our cross-sectional study found that increased dietary CoQ10 intake probably upregulated ApoA1 and downregulated TC, LDL-C, and non-HDL-C. The results of our cross-sectional study were almost consistent with the previous RCTs using relatively high doses of CoQ10 supplements in lipid regulation.

However, contrary to the result of an RCT in which supplementation with CoQ10 supplements for 24 weeks could inhibit TG levels (14), increased dietary CoQ10 intake might be accompanied by elevated TG in the current study. As we all know, diet plays a vital role in serum TG metabolism (40). Many foods provide exogenous sources of CoQ10, and since CoQ10 is fat-soluble, the concentration of CoQ10 in animal foods is higher than that in plant foods. In the current study, meat and meat products were found to be the most important dietary sources of CoQ10 intake, and oils and fats were also important dietary sources of CoQ10 intake. Excessive intake of these foods may lead to elevated serum concentrations of TG (41). We speculated that the opposite effects of dietary CoQ10 and supplement intake on TG might be due to the reduction effect of low doses of dietary CoQ10 on TG being overshadowed by the elevation effect of other food components on TG. Since food is a complex combination of multiple ingredients, it is challenging to justify this hypothesis as our study was only an observational study, and this hypothesis needed to be further verified by cohort studies in the future.

Consistent with the overall results, the associations between dietary CoQ10 intake and TC, LDL-C, ApoA1, and non-HDL-C showed similar statistical trends in different subgroups. However, the elevated TG accompanying increased dietary CoQ10 intake was only considered statistically significant among women, people aged <60, and northern people. It suggested that sex, age, and region might be potential modifiers in the associations between CoQ10 intake from dietary sources and TG.

As we can see from the RCS curves, the dietary CoQ10 intake from Q3 to Q4 showed nonlinear negative relationships with TC, non-HDL-C, and LDL-C and nonlinear positive correlations with ApoA1 and TG. However, although the trends existed, the effect sizes were small, and the rate of these increases or decreases slowed with increasing dietary CoQ10 intake. Additionally, the RCS curve indicated that HDL-C was not significantly affected by dietary CoQ10 intake. These indicated that the practical applications of lipid profile regulation through dietary supplementation with CoQ10 may be limited. Typically, doses from 100 to 900 mg/day were used in RCTs to investigate the effects of CoQ10 supplements on lipid profiles (42). In comparison, the doses of CoQ10 obtained through diet were relatively low. For example, 200 g of chicken thighs, 130 g of pork liver, or 690 g of broccoli will only give about 5 mg of CoQ10 (30). Supplementing with CoQ10 through food may mean consuming large amounts of animal viscera and meat. Considering the positive association between dietary CoQ10 intake and TG, supplementing with CoQ10 by consuming large amounts of animal foods may not be a good option. For individuals with CoQ10 deficiency or dyslipidemia, low-dose CoQ10 included in the diet alone may not be sufficient to regulate lipid profiles effectively, and supplementation with relatively high doses of CoQ10 supplements may be a better choice than CoQ10 from dietary sources in lipid regulation.

We speculate that dietary CoQ10 intake may regulate blood lipids through the following potential mechanisms. Some studies indicated that CoQ10 and cholesterol were produced via the same biosynthetic pathway, and the exogenous CoQ10 might prevent the liver from producing cholesterol by the decreased enzymatic activity of 3-hydroxy-3-methylglutaryl coenzyme A reductase (43, 44). This potential mechanism was plausible to explain our findings between increased dietary CoQ10 and decreased TC, LDL-C, and non-HDL-C. CoQ10 supplementation increased the concentrations of CoQH_2_ (the reduced form of CoQ10) in the plasma and lipoproteins (mostly LDL and VLDL), enhancing the resistance of LDL to radical oxidation and further improving atherogenesis (45–47). Additionally, previous studies from our group have shown that CoQ10 supplementation could promote HDL-mediated macrophage cholesterol efflux and the anti-inflammatory function of HDL in Chinese adults with dyslipidemia (48).

The main strength of our study was that we used nationally representative Chinese healthy adults without a diagnosis of CVDs. Secondly, our study estimated the mean dietary CoQ10 intake in the Chinese population for the first time. It focused on the benefits of CoQ10 obtained from food instead of CoQ10 supplements used in other studies, thus filling a knowledge gap on the associations between dietary CoQ10 intake and lipid profiles. Another strength was that we looked at the dose–response associations and effect modifiers.

Nevertheless, there were some limitations in this study. First, the cross-sectional study could not perform causal inference, and further cohort studies are needed in the future to confirm causality. Second, due to the difficulty of the national cross-sectional study, this study did not evaluate the level of CoQ10 in plasma. In the future, RCT with ideal and controlled circumstances should be used to better explain the relationship between dietary CoQ10 intake and blood lipid profiles.

## 5 Conclusion

In summary, this study suggested that higher CoQ10 intake from dietary sources was related to the limited ameliorative effects of TC, LDL-C, ApoA1, and non-HDL-C in general Chinese adults without a diagnosis of CVDs. However, higher CoQ10 intake from dietary sources might be associated with increasing TG, and this association might be influenced by sex, age, and region. CoQ10 from dietary sources may not be a good choice for individuals who need to supplement CoQ10.

## Data Availability

The studies involving humans is based on the CHNS, an open dataset from a national observational study. The CHNS study was carried out following the Declaration of Helsinki, and approved by the Ethics Committee of the University of North Carolina at Chapel Hill and the National Institute for Nutrition and Health, Chinese Center for Disease Control and Prevention. Written informed consent for participation was required from the participants or the participants’ legal guardians/next of kin in accordance with the national legislation and institutional requirements.
