# Supplementary material for: Associations between dietary coenzyme Q10 intake and lipid profiles in adults: a national cross-sectional study
Source: Front Nutr. 2024 Nov 12;11:1472002. doi: 10.3389/fnut.2024.1472002 (PMC11600311; doi:10.3389/fnut.2024.1472002)
Supplement: Supplementary file 1 [file Table_1.DOCX]

Achieving a framework of the circular economy in urban transport infrastructure projects: a meso-scale perspective

Xinyu Liu^1,2*^, Daan Schraven^3*^, Wenting Ma^4^, Martin de Jong^5,6^, Marcel Hertogh^1^

^1^Faculty of Civil Engineering and Geosciences, Delft University of Technology, Delft, The Netherlands

^2^Loughborough Business School, Loughborough University, Loughborough, The United Kindom

^3^Faculty of Architecture and the Built Environment, Delft University of Technology, Delft, The Netherlands

^4^School of Humanities and Social Sciences, Harbin Institute of Technology (Shenzhen), Shenzhen, China

^5^Rotterdam School of Management, Erasmus University Rotterdam, Rotterdam, The Netherlands

^6^School of International Relations and Public Affairs, Fudan University, Shanghai, China

*** Correspondence:**Corresponding Author
[x.liu@lboro.ac.uk](mailto:x.liu@lboro.ac.uk); d.f.j.schraven@tudelft.nl

Supplementary Material

# S1 Supplementary Tables

Table S1. Pro(s)/Con(s)^[[1]](#footnote-1)^ and criteria that the 17 CE “frameworks” met

| **Reference** | **Framework name** | **Pro(s)** | **Con(s)** | **Criteria met** |
| --- | --- | --- | --- | --- |
| Iacovidou et al. (2017) | CVORR framework | Multi-actor | Confined to the assessment of resources recovered from waste | ②③④ |
| Witjes and Lozano (2016) | ProBiz4CE framework | A collaborative framework between SPP (sustainable public procurement) and SBM (sustainable business model) | The framework is based on CE only by closing loops through recovery | ②③④ |
| Mendoza et al. (2017) | BECE framework | Generic and applicable across different products and business sectors; flexible; integrating top-down (business model) and bottom-up (product-service design) considerations; helping companies to develop sustainable business models that translate CE principles into industrial practice; bridging the gap between the strategic and operational levels | Complex | ①②③④ |
| (Ang et al., 2021) | PM9R framework | A systematic framework to guide the implementation of the CE concept | Confined to the pharmaceutical manufacturing (PM) industry | ①② |
| (Çetin et al., 2021) | CDB framework | Provides a fruitful starting point for the novel research avenue at the intersection of CE, digital technology and the built environment, and gives practitioners inspiration for sustainable innovation in the sector | Confined to digital technologies | ②④ |
| (Coenen et al., 2020) | CEIMA framework | Connects infrastructure stakeholders to concrete applications of CE through identification of possible interfaces; based on the “9R” waste hierarchy, actions are formulated that provide a practical guide to more circular infrastructure | CE actions are limited | ①②③④ |
| Winning et al. (2017) | global ENGAGE-materials model | Can address both upstream and downstream impacts of resource efficiency and the CE policy implications | At a macro-economic and sectoral level | ③ |
| (Muranko et al., 2018) | P-CCM framework | Supporting Pro-Circular behaviors | Confined to technical goods | ① |
| (van Stijn et al., 2021) | CE-LCA model | Supports an ex-ante assessment of circular building components in a theoretical context | Time-consuming to use | ②③④ |
| (Sinclair et al., 2018) | Consumer Intervention Mapping tool | Can create scenarios that describe existing product service systems and new product concepts adapted to a CE paradigm | Confined to Product Service Systems | ①③④ |
| (Álvarez and Ruiz-Puente, 2017) | SymbioSyS tool | Helps promote the efficient use of resources and new business models through the detection of the main synergies among companies based on the substitution of raw materials from waste, sub-products, or recycled materials, and the possibility of new opportunities for collaboration in the sharing of infrastructure and services | Mainly lies in industrial ecology (IE) | ①③④ |
| (Lauten-Weiss and Ramesohl, 2021) | CBF tool | An extensive guide for structuring business models (BMs) | Confined to business case | ②③④ |
| (Ferronato, 2021) | NAVA-CE approach | Supporting solid waste management development projects | Confined to low to middle-income countries | ③ |
| (van Bueren et al., 2023) | “eco Quintuple Helix Model” (eco-5HM) | can better inform decision-makers  for CE operations and tactics to achieve the strategic goal of sustainability | Would not guide to a more limited scope of tactics and operations | ②③ |
| (D’Urzo and Campagnaro, 2023) | Design-led Repair & Reuse (DLRR) framework | generates a higher quality of processes and products from circular, low  entropy and low capital-intensity production activities | Confines in the reuse market | ①②③ |
| (Wiprächtiger et al., 2023) | IE4CE approach | combines Industrial ecology (IE) methodologies to determine the environmental impact mitigation potential of CE strategies for a defined geographic region | the design of the scenarios is associated with many uncertainties | ③ |
| (Diacono and Baldacchino, 2023); (Sadeghi et al., 2023)^[[2]](#footnote-2)^ | ReSOLVE framework | e.g., a useful framework to advance knowledge on circular entrepreneurship | e.g., Opportunities evaluation and exploitation were not explored | ①②③④ |

Table S2. The “4Wh-iReSOLVE” analytical framework for urban transport infrastructure projects*

| **4wh** | **Specification:** | | **Environmental impact (total)** | **Economic impact (total)** | **Social impact (total)** |
| --- | --- | --- | --- | --- | --- |
| ***Who:***  Stakeholders | 1. Public agency 2. Contractor 3. …   ...   1. … | | ... | ... | ... |
| ***When:***  Life cycle stages | - (D/P) Design/preparation - (C) Construction - (O/M) Use and Maintenance - (D/D) Decommissioning and demolition | | ... | ... | ... |
| ***Where:***  Summary of specific locations and attributes | 1. Roof 2. Off-site factory 3. …   ...   1. … | | ... | ... | ... |
| ***What:***  **iReSOLVE Actions** | **iReSOLVE requirement activities** | ***Example specification (who, when, where) **** | **Environmental impact (per action)** | **Economic impact (per action)** | **Social impact (per action)** |
| Regenerate | Shift to renewable energy and materials | *use of bioplastics (2, C, ii)* | Long or Short term | N/A | N/A |
|  | Reclaim, retain, restore health of ecosystems | *promote restoration of grasslands (2,D/D,…)* | N/A | Long or Short term | N/A |
|  | Return recovered biological resources to biosphere | *N/A* | ... | ... | ... |
| Share | Share assets | *share appliances (2,C,…)* | … | ... | ... |
|  | Reuse, second-hand use | *biofuels derived from used cooking oil (2,C,…)* | ... | ... | ... |
|  | Prolong product life (durable, design, maintenance, repair) | *easy to clean filters (…,…,…)* | ... | ... | ... |
| Optimise | Increase product performance and efficiency | *use engines with improved fuel efficiency (2,C,…)* | ... | ... | ... |
|  | Remove waste in production and supply chain |  | ... | ... | ... |
|  | Leverage big data, automation, remote sensing, steering |  | ... | ... | ... |
| Loop | Remanufacture products or components | *label repairable/recyclable/elements (…,…,…)* | ... | ... | ... |
|  | Recycle materials | *increase recycled content and recyclability (…,…,…)* | ... | ... | ... |
|  | Digest anaerobically | *N/A* | ... | ... | ... |
|  | Extract biochemical from organic waste | *N/A* | ... | ... | ... |
| Virtualize | Dematerialize directly | *remove the wiring system cordless (…,…,…)* | ... | ... | ... |
|  | Dematerialize indirectly | *online shopping (…,…,…)* | ... | ... | ... |
| Exchange | Replace old with advanced non-renewable materials** |  | ... | ... | ... |
|  | Apply new technologies | *3D printing (2,C,…)* | ... | ... | ... |
|  | Choose new products/services | *use graphene (…,…,…)* | ... | ... | ... |
| Implement | An ambitious vision/target | *consumption of energy, water and materials minimized (2,C,…)* | ... | ... | ... |
|  | A scaled-up business plan with a roadmap |  | ... | ... | ... |
|  | Stakeholder engagement | *government authorities (…,…,…)* | ... | ... | ... |
|  | Systems thinking | *value chain, cross-cycle, cross-sector (…,…,…)* | ... | ... | ... |
|  | Specific step-by-step guidelines and supporting tools |  |  |  |  |

*according to Mendoza et al. (2017); the phrases in parentheses and italics are examples of the corresponding “iReSOLVE requirements”

** We doubt that the “non-renewable” here in the original table should be “renewable”

Table S3. The overall list of CE activities of Project 1 to 10

| No. | (D/P) (C) |
| --- | --- |
| 1.1 | harvest all materials from the viaducts to be demolished in the A76 (p4) |
| 1.2 | The baffle plates and girders, including railings from the Holeweg viaduct, are also reused (p21) |
| 1.4 | the foundation consists of recycled steel tubular piles (p4) |
|  | (D/P) |
| 1.6 | Strategic sessions with administrators for further scaling up and further development of the CTL concept (p11) |
| 1.8 | The connections are designed in such a way that any future disassembly is possible (p26) |
| 1.12 | Interviewing managers within and outside Rijkswaterstaat to gain insight into demolition plans of existing structures (p11) |
| 1.13 | Develop and determine the content of the "reusability scan" together with (knowledge) partners and stakeholders (p11) |
| 1.14 | Testing and further development of the design concepts with knowledge partners and knowledge institutions (TNO and TU Delft) (p12) |
| 1.15 | Testing the feasibility of the parts to be harvested together with Lek Sloopwerken (p12) |
| 1.16 | Prior to the application of the 'reusability scan', the content of the tool was determined together with the (knowledge) partners (p14) |
|  | (O/M) |
| 1.7 | regular maintenance of the parts to be reused is substantiated (p26) |
|  | (D/P) (O/M) |
| 1.9 | production of circular concrete and asphalt (p28) |
|  | (D/D) |
| 1.3 | 72.5% of the existing viaduct components can be reused in a high-quality manner (p5) |
| 1.5 | removal of the asphalt for high-quality recycling (p24) |
|  | (D/P) (C) (O/M) (D/D) |
| 1.10 | Knowledge sharing within and outside our consortium (p6) |
| 1.11 | knowledge sessions and dialogues with administrators and market parties (p9) |
| 1.17 | bring supply and demand together and guarantee that the concept is suitable for every organization and customer (p29) |
| No. | (D/P) (C) |
| 2.1 | use the beams released at the Kromwijkdreef (p10) |
|  | (C) |
| 2.2 | Reuse of physically unaltered inverted T-beams (p17) |
|  | (D/P) |
| 2.5 | We are introducing our platform www.liggerbank.nl to better match market-wide supply and demand (p2) |
| 2.6 | to better coordinate market-wide questions and answers and are continuing with the database to detect reusable beams from the area of viaducts to be demolished (p16) |
|  | (D/P) (D/D) |
| 2.7 | We are leading the way in establishing a non-exclusive, public marketplace to connect supply & demand (p3) |
|  | (D/P) (C) (O/M) (D/D) |
| 2.3 | make our acquired knowledge, insights and experiences available to the market free of charge (p3) |
| 2.4 | A large storage capacity is also secured via Dura Vermeer Urban Miner. The consortium works in the same project environment, so that all information is available to everyone. (p5) |
| No. | (D/P) (C) |
| 3.1 | The concrete arch construction ensures efficient power transfer and makes impact plates unnecessary (p3) |
| 3.2 | no joint transitions and bearing blocks are required (p3) |
| 3.3 | maintenance-sensitive parts such as joint transitions, bearing blocks and kick plates are not required (p20) |
| 3.5 | a green connection makes a positive contribution to defragmentation of natural areas and increasing biodiversity (p15) |
| 3.6 | All elements can be disassembled and have standardized dimensions and connections. This makes the system reusable and interchangeable on element level (p3) |
| 3.7 | a fully recoverable, modular arch construction and substructure (p2) |
| 3.8 | detachable connections of the arch construction and the recoverable, demountable substructure (p3) |
| 3.9 | The soil backfill to the superstructure results in completely releasable connections to the road surfacing, rainwater drainage and sheathing pipes for cables and pipes. This provides a high degree of flexibility and adaptability, but also offers advantages in terms of management and maintenance (p25) |
|  | (D/P) (C) (O/M) (D/D) |
| 3.4 | The parties involved complement each other to form a complete and integral team with all the necessary expertise in the field of technology, sustainability and entrepreneurship. We work shoulder to shoulder from one consortium to one common goal: a maximum reduction in the use of primary raw materials for viaducts (p3) |
| 3.10 | we use the knowledge and skills of all team members and the underlying organizations (p7) |
| 3.11 | we evaluated our cooperation every month and adjusted it where necessary (p7) |
| 3.12 | We make the right to use our innovation available to everyone (p31) |
| 3.13 | all parties have access to the circular construction system and the entire sector can contribute to scaling up (p31) |
| No. | (D/P) (C) |
| 4.1 | Substantial material reduction by applying UHSVB in the ultimate bend-free shape (p5) |
| 4.2 | the arch shape from UHSVB saves volume and the geopolymer concrete saves cement and new aggregates (p18) |
| 4.3 | the design with UHSVB has been optimized so that less material is needed during construction (p19) |
| 4.5 | pressure lines are pushed closer together, the bending is reduced and therefore also the use of materials (p21) |
| 4.6 | The Circular Arch Viaduct is an integral bridge in which the construction is mechanical as much as possible coupled with foundation piles. This is material efficient and allows easy disassembly (p23) |
|  | (D/P) |
| 4.4 | integral bridges have no supporting devices, expansion joints that require a lot of maintenance and where problems often arise that have a negative impact on the overall lifespan of the construction (p5) |
| 4.7 | the Circular Arch Viaduct lends itself perfectly as an ecoduct to allow fauna to pass through (highway) roads (p17) |
| 4.13 | a concrete mixture based on a 100% secondary granular skeleton in combination with a binder free of Portland cement (= 100% circular geopolymer concrete) (p24) |
| 4.14 | a geopolymer concrete with 100% secondary skeleton (recycled sand and granulate) (p24) |
| 4.15 | A processable geopolymer concrete with 100% recycled material for application in the abutment (p25) |
| 4.21 | With an international contractor, international engineering firm, prefab concrete producer and specialist knowledge of UHSVB and geopolymer concrete in our consortium, we are able to scale up innovation in the Netherlands and beyond (p6) |
| 4.22 | The length of the arch beams can be varied to make the dimensions suitable for each project. The arch beams will be in a number of standard sizes come onto the market, so that they are also interchangeable between different projects (p29) |
|  | (D/P) (D/D) |
| 4.8 | reusable foundation piles (p5) |
|  | (D/D) |
| 4.9 | Abutments of circular and cementless geopolymer concrete where granulate from old viaducts as 100% recycled building materials are reused (p5) |
| 4.10 | The UHSVB (Ultra High Strength Fiber Reinforced Concrete) elements can be reused during their longer lifespan (p19) |
| 4.11 | The UHSVB elements and steel tubular piles are reused during a life cycle (p19) |
| 4.12 | A modular, demountable and reusable viaduct of UHSVB elements (p5) |
| 4.16 | regularly screw out the tubular piles for reuse (p26) |
|  | (D/P) (C) (D/D) |
| 4.17 | a connection has been developed that makes the pile foundation detachable from the abutments, in order to recover the tubular piles after the lifespan of the Circular Arc Viaduct (p11) |
|  | (D/P) (C) (O/M) (D/D) |
| 4.18 | The results are published online under the public open-source license CC-BY-SA of Creative Commons (p27) |
| 4.19 | To ensure fitability for future projects, we hold an (online) seminar for project teams of clients to take into account the adaptability of the Circular Arch Viaduct (p27) |
| 4.20 | For the further dissemination of the ideas in the Rijkswaterstaat organization, we use the network of the SBIR Call for Circular Viaducts Project Team to organize lectures and inform project teams (p27) |
| No. | (D/P) (C) |
| 5.1 | Application of wood (p2) |
| 5.2 | All secondary elements such as the edge elements, guide rails and parts of the substructure are made of circular building materials such as European wood (p8) |
| 5.3 | Slim steel deck sections; with which we achieve a large weight reduction (p2) |
| 5.4 | The deck sections have a closed cell structure, creating a relatively lightweight solution (p8) |
| 5.5 | using a steel bridge deck and hollow foundation elements (p12) |
| 5.9 | The Variaduct consists of light, remountable building elements that are easily reusable (p8) |
|  | (D/P) |
| 5.6 | Foundation without piles; the light construction makes it possible to omit pile foundations in 9 out of 10 cases (p2) |
| 5.7 | Due to a large reduction in its own weight (73%) (compared to the reference viaduct), a pile foundation is not necessary (p3) |
| 5.8 | Reduction of the construction height; thus limiting material (p2) |
| 5.12 | a circular, modular and remountable design for the substructure (p4) |
| 5.13 | We can extend the Variaduct or replace parts while preserving the existing construction and foundation. As a result, the Variaduct will not have to be demolished in the future before the end of the technical life, which is often the case now (p8) |
| 5.14 | all interfaces and connections have been designed according to IFD (Industrial, Flexible and Demountable Building) (p16) |
| 5.15 | The elements are mutually connectable with a bolt connection. This connection is durable and easy to disassemble (p18) |
| 5.16 | An angled abutment with joint construction is, of course, removable and applicable everywhere (p19) |
|  | (D/P) (D/D) |
| 5.11 | the Variaduct can be disassembled and then reassembled |
|  | (D/P) (C) (O/M) (D/D) |
| 5.10 | In addition to settlement behaviour, we monitor the traffic intensity with a traffic counter. We process this information in the materials passport and use it, among other things, to increase the reusability of the building elements (p15) |
| 5.18 | The open source approach of the Variaduct ensures that many parties can use it (p4) |
|  | (C) (O/M) (D/D) |
| 5.17 | the use of electrical equipment, clean fuel and the use of NOX filters (p3) |
| No. | (C) |
| 6.1 | a pressure-optimized construction that makes very efficient use of the material (p17) |
|  | (D/P) (C) |
| 6.2 | Additive manufacturing (AM): material is only placed where it is needed and cutting losses and auxiliary structures such as formwork are prevented (p3) |
| 6.3 | AM does not require mold material (p24) |
| 6.11 | Applying improved and new low-CO_2_ (concrete) mixtures (p3) |
| 6.12 | efficient light spatial structure of 3D-printed concrete (p2) |
| 6.17 | Digital supply chain: design, production and realization take place in a digital supply chain (p3) |
|  | (D/P) |
| 6.4 | Parametric and Optimized Designs: This leads to scalable designs and a strong reduction of material use (up to 60%) (p3) |
| 6.5 | The arch construction is composed of blocks with a light hollow structure (p4) |
| 6.22 | The concept is ideal for combining with other circular partial solutions (p7) |
|  | (O/M) |
| 6.16 | Broken or damaged parts can be replaced quickly and cheaply (p12) |
|  | (D/P) (D/D) |
| 6.6 | the raw materials used are 100% reusable in a second phase of life and beyond (p12) |
| 6.10 | Waste-free production process (no moulds) (p12) |
| 6.14 | Modular and detachable design: the printed blocks are joined together using prestressing techniques (p3) |
| 6.15 | composing the viaduct from modular elements (approx. 80%) (p7) |
| 6.7 | The 'manual for reuse' contains everything for the next generations: the specifications, the different ways in which the object can be dismantled and how the building blocks can be reused according to the current insights and standards (p24) |
|  | (D/P) (C) (O/M) (D/D) |
| 6.8 | The knowledge that is shared through user groups concerns the specifications, requirements and design aspects that the building blocks must meet in order to guarantee a safe construction and to encourage reuse (p29) |
| 6.9 | The Format 'Manual for reuse' is freely available (p29) |
| 6.13 | A service market instead of a producing market (p24) |
| 6.18 | constant focus on our shared ambition and attention to each other's interests (p11) |
| 6.19 | The fascination for the opportunities of 'digital concrete' for circular construction is an important shared motivation (p11) |
| 6.20 | Through dedication to the subject and the partners' complementary business models, the collaboration runs very smoothly. This underlines the intrinsic motivation of the parties. They are all authorities on their own expertise and knowledge is shared as much as possible (p26) |
| 6.21 | Knowledge is shared reciprocally in user groups (p28) |
| No. | (D/P) (C) |
| 7.1 | a completely wooden superstructure on a conventional substructure (p15) |
| 7.7 | all non-replaceable load-bearing parts are protected, standing water on the bridge is prevented, the side is protected against rain impact, the elements are ventilated and inspectable (p17) |
| 7.8 | The deck and a waterproof membrane protect the main beams and prevent rain from falling onto the beams from above (p18) |
| 7.9 | ventilation around the main girders and the ventilation between deck and main girder formed by the transverse beams. If water does come through the deck, there is no direct surface contact with the main beams (p18) |
|  | (D/P) (C) (O/M) |
| 7.10 | The detachability of the cover elements serves to change the elements in unexpected situations and to protect the main carrying system (p17) |
|  | (D/P) (C) (D/D) |
| 7.11 | The simplicity of the construction and the releasable connections make the assembly and disassembly method easy (p21) |
|  | (D/P) (D/D) |
| 7.2 | replaceable decking elements for reuse (p16) |
| 7.3 | The first priority in reuse is to use the beams as beams in a subsequent viaduct (p24) |
| 7.4 | Another use is as a beam with lesser load, such as a bicycle/pedestrian bridge (p24) |
| 7.5 | Re-use outside the original use is sawing the girders into trusses or purlins in roof constructions for large spans (p24) |
| 7.6 | the wood can be used as sawn planks and beams or as input for derived wood products (p24) |
|  | (D/P) (C) (O/M) (D/D) |
| 7.12 | Mutual reviews resulted in an exchange of knowledge and information (p4) |
| No. | (D/P) (C) |
| 8.4 | The cover plates are made of UBB. By the application of this high-strength concrete and the industrially standardized fabrication, a concrete surface is created of very high quality that is extremely durable. The concrete is resistant to freezing and de-icing salts and no sealing (such as hydrophobizing agent and/or asphalt) needs to be applied (p14) |
|  | (D/P) |
| 8.1 | we generate our own renewable energy (wind and solar energy), with which we operate our prefab factories supplied with green energy (p10) |
| 8.2 | We have developed the environmentally friendly high-strength geopolymer concrete Umweltbeton Bögl (UBB) (named GPB 2.0 in the project proposal) for our cover plates (p6) |
| 8.3 | Mageba has developed modular bearing blocks made of corrosion-free steel (p8) |
| 8.5 | The steel beams are manufactured in standard sizes of 39.90 m, 31.92 m, 26.60 m and 21.28 m. All these dimensions are a multiple of 2.66 m which is the standard width of our cover plate. As a result, there are no fitting plates required and each cover plate is interchangeable (p13) |
| 8.15 | abutments of building blocks made of UBB, or recycled concrete (p8) |
| 8.17 | We have further elaborated our vision on the application of the All-in consortium (p9) |
| 8.18 | All parts of the MVB are modular and circular (p3) |
| 8.19 | By means of a steel sleeper between the longitudinal beams we have a detachable connection with the abutment created (p8) |
| 8.20 | We have developed a modular joint transition in collaboration with Mageba. This joint transition is bolted to the abutment side and to the cover plate side and can be dismantled without cutting and breaking (p8) |
|  | (D/P) (D/D) |
| 8.16 | After the service life of 200 years, the beams can be melted down into new steel products (p13) |
|  | (D/P) (C) (D/D) |
| 8.7 | The pillar and base are linked together, creating a single element that can be disassembled and reused (p8) |
| 8.8 | At the bottom, the pillars are attached to the base, whereby the base and the pillars form one modular element. This element can be disassembled and reused as a whole. This makes the pillars removable and reusable (p13) |
| 8.10 | The building blocks can be easily disassembled and subsequently reused (p12) |
| 8.11 | The concrete L-walls and building blocks are produced in standard sizes, are demountable and reusable (p13) |
| 8.12 | The cover plates are fitted perpendicular to the direction of travel on the support strips of the steel girders and post-tensioned to form a compact deck. As a result, the deck and the seams between the deck plates are watertight and no sealing and asphalt need to be applied. This makes the cover plates removable and reusable (p14) |
| 8.14 | The distribution of the load on the bearing blocks on one support point can be checked and adjusted so that tolerances are eliminated. The modular bearing blocks are removable and reusable (p15) |
|  | (D/P) (C) (O/M) |
| 8.13 | The joint transition only uses removable, reusable components and materials, is maintenance-free (p14) |
|  | (D/P) (C) (O/M) (D/D) |
| 8.6 | all MVB components are modular and circular with a reduced CO_2_ emissions and minimal material consumption (p3) |
| 8.21 | The data collected by us is made available as open source (p9) |
| 8.22 | we grant the right to use our innovation free in the Netherlands (p9) |
| 8.9 | All MVB components are removable and reusable; The building blocks can be easily disassembled and subsequently reused (p10) |
| No. | (D/P) (C) |
| 9.1 | The prefab beam has a width of 1.6 meters and consists of ¼ part of recycled concrete and ¾ part of European spruce. The wood is protected against weather influences by the concrete (p3) |
| 9.5 | Instead of cement, we (re)use industrial by-products as a binding agent (p6) |
| 9.6 | Our beam design is suitable for reusing existing abutments and foundations, provided the function of the viaduct to be replaced remains the same. Due to the weight reduction of the deck construction, it is plausible that existing abutments and foundations are sufficient (p7) |
| 9.12 | With a thoughtful design of the concrete cover layer and edge element, the wood is protected from moisture and other weather influences (p7) |
| 9.13 | We protect the ends of the wood at the abutments with a solid concrete headboard (p11) |
| 9.14 | To protect the wooden beam on the outside of the viaduct against weather influences, it has been chamfered in the shape of a staircase and placed under the edge element. This means that no direct rainwater can reach the edge girders (p11) |
| 9.15 | the wooden beams are completely shielded on the top, longitudinal and end sides due to the concrete deck design (p13) |
| 9.18 | We finish the concrete surface with a waterproof layer, for example a (prefab) bituminous membrane (p11) |
|  | (D/P) |
| 9.2 | a new tree is planted for every tree felled for forest conservation (p7) |
| 9.9 | Recycled concrete made with secondary raw materials such as recycled sand, gravel and/or concrete granulate (p7) |
| 9.10 | The concrete is made of recycled concrete or geopolymer concrete (p10) |
| 9.16 | Our design has a detachable center support (p7) |
| 9.17 | We provide the beams with a waterproof coating and hardening (p10) |
|  | (D/P) (D/D) |
| 9.3 | The circular value of the products and materials used at the end of their technical life offers opportunities for high-quality reuse, also outside civil engineering (p6) |
| 9.4 | After the viaduct's functional lifespan (average 43.6 years), the girders can be reused in a new viaduct to be built (p6) |
| 9.7 | Our wood-concrete design has a modular structure on 4 levels. With this we achieve high-quality reuse after the first use phase (p9) |
| 9.8 | Steel tubular piles can be recovered for reuse (p11) |
|  | (O/M) |
| 9.11 | Using new techniques, the sensors generate data and send it via the Internet of Things to intelligent systems (p13) |
|  | (C) (O/M) |
| 9.19 | By spacing the wooden beams there is enough space to inspect the individual beams (p13) |
| No. | (D/P) |
| 10.1 | Replacing the glass fibers with flax fibers (p7) |
|  | (D/P) (D/D) |
| 10.2 | The advantage of a geotextile is that it can be completely removed and reused at another location. Most textiles are already made from (partly) recycled material (p5) |
|  | (D/P) (C) (O/M) (D/D) |
| 10.3 | By sharing our acquired knowledge open source (via a website), we share our acquired knowledge free of charge for other parties to include this innovation in their products catalogue. This increases awareness among clients and administrators (p4) |
| 10.4 | constructive interaction between RWS, knowledge institutions and the experts (p5) |
|  | (D/P) (C) |
| 10.5 | light weight biobased composite (p1) |

iReSOLVE requirement activities based on different lifecycle phases of Project 1-10 (with page number information according to the original reports of Project 1-10) [note: Design/preparation: (D/P), construction: (C), use and maintenance: (O/M), decommissioning and demolition: (D/D)]

Table S4. CE activities of Project 1-10 based on iReSOLVE action categories (the numbers are in correspondence with those in Appendix Table S3; the numbers in italics mean the corresponding CE statements belong to more than one sub-category)

| R: Regenerate | R1 Shift to renewable energy and materials (e.g. Use of bioplastics) | 5.1, 5.2,  7.1,  8.1,  9.1, |
| --- | --- | --- |
|  | R2 Reclaim, retain, restore health of ecosystems (e.g. Promote restoration of grasslands)) | 3.5,  4.7,  9.2, |
|  | R3 Return recovered biological resources to biosphere |  |
| S: Share | S1 Share assets (e.g. Share appliances)) |  |
|  | S2 Reuse, second-hand use (e..g. Biofuels derived from used cooking oil)) | 1.2, *1.3*,  2.1, 2.2,  3.6,  4.22, 4.8, 4.9, 4.10, 4.11, 4.12, 4.16,  5.9, 5.10,  6.6, 6.7, 6.8, 6.9,  7.2, 7.3, 7.4, 7.5, 7.6,  8.7, 8.8, 8.9, 8.10, 8.11, *8.12*, 8.13, 8.14,  9.5, 9.6, 9.3, 9.4, 9.7, 9.8,  10.2, |
|  | S3 Prolong product life (durable design, maintenance, repair) (e.g. Easy-to-clean filters) | *1.3*, 1.7, 1.8,  3.7, 3.8, 3.9,  4.17,  5.12, 5.13, 5.14, 5.15, 5.16, 5.11,  6.16, 6.14, 6.15,  7.7, 7.8, 7.9, 7.10, 7.11,  *8.4*, *8.12*, 8.18, 8.19, 8.20,  9.12, 9.13, 9.14, 9.15, 9.18, 9.16, 9.17, 9.19, |
| O: Optimize | O1 Increase product performance and efficiency (e.g. Use engines with improved thermal, fuel efficiency) | 6.1,  8.2, 8.3, |
|  | O2 Remove waste in production and supply chain | 6.10, |
|  | O3 Leverage big data, automation, remote sensing, steering | 6.17, |
| L: Loop | L1 Remanufacture products or components (e.g. Label reparable/upgradable elements) |  |
|  | L2 Recycle materials | 1.1, 1.4, 1.5,  4.13, 4.14, 4.15,  8.15, 8.16,  9.9, 9.10,  10.2, |
|  | L3 Digest anaerobically |  |
|  | L4 Extract biochemical from organic waste |  |
| V: Virtualize | V1 Dematerialize directly (e.g. Remove the wiring system (cordless)) | 3.1, 3.2, 3.3,  4.1, 4.2, 4.3, 4.5, 4.6, 4.4,  5.3, 5.4, 5.5, 5.6, 5.7, 5.8,  6.2, 6.3, 6.12, 6.4, 6.5,  *8.4*, 8.5 |
|  | V2 Dematerialize indirectly (e.g. online shopping) |  |
| E: Exchange | E1 Replace old with advanced renewable materials | 10.1, |
|  | E2 Apply new technologies (e.g. 3D printing) | 6.11,  9.11, |
|  | E3 Choose new products/services (e.g. multimodal transport) | 1.9, |
| I: Implement | I1 An ambitious vision/target (e.g. The consumption of energy, water and raw materials minimized as much as possible) | 3.4,  8.6, |
|  | I2 A scaled-up business plan with a roadmap | 1.6,  *4.21*,  6.13,  8.17, |
|  | I3 Stakeholder engagement | *1.10, 1.11, 1.12*, 1.13, 1.14, *1.15*, 1.16, *1.17*,  2.3, 2.4,  3.10, 3.11, 3.12, 3.13,  *4.21*, 4.18, 4.19, 4.20,  5.18, 5.17,  6.18, 6.19, 6.20, 6.21,  7.12,  8.21, 8.22,  (*10.3*), (10.4), 10.5 |
|  | I4 Systems thinking (value chain, cross-cycle, cross-sector) | *1.10, 1.11, 1.12*, *1.15, 1.17*,  *2.5*, 2.6, 2.7,  6.22,  (*10.3*) |
|  | I5 Specific step-by-step guidelines and supporting tools | *2.5*, |

1. In respect to the focus of this paper [↑](#footnote-ref-1)
2. These two papers both just used the established ReSOLVE framework so the pro(s) and con(s) here are examples. [↑](#footnote-ref-2)
